# MiR-106b-5p regulates the migration and invasion of colorectal cancer cells by targeting FAT4

**DOI:** 10.1042/BSR20200098

**Published:** 2020-11-02

**Authors:** Min Pan, Qiuqiu Chen, Yusong Lu, Feifei Wei, Chunqiao Chen, Guiyan Tang, Hui Huang

**Affiliations:** Department of Oncology and Radiotherapy, Guilin People’s Hospital, Guilin, China

**Keywords:** Colorectal cancer, FAT4, MicroRNA-106b-5p

## Abstract

MicroRNA-106b-5p (miR-106b-5p) is involved in the development of many cancers including colorectal cancer (CRC), and FAT4 is correlated with regulation of growth and apoptosis of cancer cells. The present study aimed to investigate the relation between FAT4 and miR-106b-5p and the underlying mechanism of the two on the development of CRC. Quantitative real-time PCR (qRT-PCR) assay and Western blot (WB) analysis were performed to detect the expressions of messenger RNAs (mRNAs), microRNAs (miRNAs) and proteins. The viability of CRC cells was detected by cell counting kit-8 (CCK-8). Scratch test and transwell assay were performed to measure the migration and invasion of CRC cell. Tumor angiogenesis was simulated by *in vitro* angiogenesis experiment. Dual-luciferase reporter assay was performed to verify the targeting relation between miR-106b-5p and FAT4. The study found that the expression of FAT4 was down-regulated and that of miR-106b-5p was up-regulated in CRC tissues. Overexpression of FAT4 resulted in decreased proliferation, migration, invasion and angiogenesis of CRC cells, whereas silencing of FAT4 led to the opposite results. In rescue experiment, miR-106b-5p partially reversed the function of FAT4 in CRC cells, thus playing a carcinogenic role by targeting FAT4 in the CRC cells.

## Introduction

Colorectal cancer (CRC) still remains one of the main causes of cancer-related mortality [1–3]. CRC cases are expected to increase by 60% by 2030, with over 2.2 million new cases and over 1.1 million CRC-caused deaths [4]. Poor prognosis of CRC patients is associated with tumor metastasis [5]. In recent years, molecular metastasis at transcriptional level and changes in microenvironment markers has attracted much research attention. Collaborative development of biomarker drugs could inhibit the development of tumors including CRC [6], thus, further research on the molecular basis of progression and metastasis of CRC may help design new drugs for the cancer treatment.

FAT4, which is a member of the cadherin gene superfamily [7], participates in the planar pathway of cell polarity and encodes adipose atypical cadherin, and FAT4 is known as one of the four human homologs of *Drosophila* adipose tissues [8]. It was reported that expression of FAT4 is low-expressed in gastric cancer [9], endometrial cancer [10] and hepatocellular carcinoma [11]. A previous study found that overexpression of FAT4 promotes cell cycle, proliferation, invasion and migration of certain cancers and inhibits tumor cell apoptosis [12]. However, the role and mechanism of FAT4 in CRC are less reported.

MicroRNAs (miRNAs) are non-coding RNAs that affect the stability of messenger RNA (mRNA) as negative regulators of protein translation, and regulate many signaling pathways and cellular processes to participate in intercellular communication [13,14]. Many miRNAs affect invasion and migration of cancer cells through directly regulating the inactivation of mRNA or the expressions of downstream effector molecules [15,16]. As FAT4 and miRNAs could affect the proliferation and migration of tumor cells, the current study aimed to determine the specific miRNA regulating FAT4 expression in CRC.

In this research, we explored the role and underlying mechanism of FAT4 in proliferation, migration and invasion of CRC cells, hoping to provide theoretical basis for CRC treatment.

## Materials and methods

### Patient samples

Fifty patients who were diagnosed with CRC from 2018 to 2019 in Guilin People’s Hospital were selected as the research subjects. The CRC tissues and paired adjacent tissues from these patients were then collected. All the tissue samples were fixed by formalin and paraffin-embedded. The current study was approved by the Ethics Committee of Guilin People’s Hospital Ethics Committee (approval number: SH20185665). The written informed consents were signed by all patients.

### Cell culture

Human normal colon cell CCD-18Co and CRC cell line (LS174T, LOVO, HT29, HCT116 and SW-620) were purchased from American Type Culture Collection (ATCC, Manassas, Virginia, U.S.A.) and these cells were cultured in RPMI-1640 medium containing 10% fetal bovine serum (FBS; Gibco, U.S.A.) at 37°C with 5% CO_2_ in a humidified incubator.

### Cell transfection

The cells were transfected with FAT4 siRNA and pc-DNA3.1-FAT4 plasmid (Shanghai Sangon Biotech, Shanghai, China). The primers were as follows: SiNC, 5′-GCGCGATAGCGCGAATATA-3′; pcNC sense 5′-UUCUCCGAACGUGUCACGUTT-3′, and pcNC antisense 5′-ACGUGACACGUUCGGAGAATT-3′; Scramble, 5′-TTCTCCGAACGTGTCACGT-3′; miR-106b-5p mimics, 5′-TAAAGTGCTGACAGTGCAGAT-3′; miR-106b-5p inhibitor, 5′-ATCTGCACTGTCAGCACTTTA-3′. The cell transfection was performed using the Lipofectamine 2000 Kit (Invitrogen, Carlsbad, CA). The cells were cultured in an incubator with 5% CO_2_ at 37°C for 4 days and prepared for further experiment.

### Grouping

To investigate the function of FAT4 in CRC, the cells were divided into control group (untreated cells), siNC (cells transfected with siNC), pcNC group (cells transfected with pcNC), siFAT4 (cells treated with FAT4 siRNA), and pcFAT4 group (cells treated with pc-DNA3.1-FAT4 plasmid). Moreover, to further explore the effects of miR-106b-5p and FAT4 on the CRC cells, the cells were divided into Scramble+pcNC (cells transfected with scramble and pcNC), siNC group (cell were transfected with scramble and siNC), mimics+pcNC (cells transfected with miR-106b-5p mimic and pcNC), inhibitor+siNC group (cells transfected with miR-106b-5p inhibitor and siNC), Scramble+pcFAT4 (cells transfected with scramble and pc-DNA3.1-FAT4 plasmid), siFAT4 (cells transfected with scramble and FAT4 siRNA), mimics+pcFAT4 (cells transfected with miR-106b-5p mimic and pc-DNA3.1-FAT4 plasmid), and inhibitor+siFAT4 group (cells transfected with miR-106b-5p inhibitor and FAT4 siRNA).

### The quantitative real-time PCR analysis

Total RNAs were extracted using TRIzol reagent (Invitrogen, Carlsbad, CA, U.S.A.). First-strand DNA was synthesized from 2 μg of total RNAs for the detection of expressions of miRNAs. Then the real-time PCR was performed by the SYBR Green PCR method (QPS-201; Toyobo) using All-in-One miRNA qPCR Detection kit (GeneCopoeia, Rockville, MD, U.S.A.). U6 was used as an internal control. The reaction condition was as follows: at 95°C for 10 min, 40 cycles at 95°C for 10 s, and at 60°C for 1 min. For detection of expressions of mRNAs, 2 μg of total RNAs were reverse-transcribed into cDNAs using a PrimeScript RT reagent kit (Takara Bio, Kyoto, Japan) and the expressions were measured by the SYBR Premix ExTaq™ II (Takara Bio, Kyoto, Japan). GAPDH served as an internal control. The reaction condition was as follows: at 95°C for 10 min, and 40 cycles at 95°C for 15 s, at 60°C 15 s. Sequences of all primers were listed in Table 1. The relative expression levels of miRNA and mRNAs were calculated by 2^−ΔΔ*C*_t_^ method [17]. The experiment was performed in triplicate.

**Table 1:** Sequences of primers used for qRT-PCR analysis

Gene	Forward (5′–3′)	Reverse (5′–3′)
*FAT4*	TATCACAAAACGCCCTTGCT	TGGATTGTCATTGATATCCTG
*VEGF*	CGAAACCATGAACTTTCTGC	CCTCAGTGGGCACACACTCC
*E-Cadherin*	TGCCCAGAAAATGAAAAAGG	GTGTATGTGGCAATGCGTTC
*N-Cadherin*	CCATCACTCGGCTTAATGGT	ACCCACAATCCTGTCCACAT
*Vimentin*	GACAATGCGTCTCTGGCACGTCTT	TCCTCCGCCTCCTGCAGGTTCTT
*GAPDH*	TGCCAAATATGACATCAAGAA	GGAGTGGGTGTCGTCGCTGTTG

### Cell counting kit-8 assay

The cells (1 × 10^5^ cells/well) were seeded into 96-well plates. Cell counting kit-8 (CCK-8) reagent (10 ml) was added to each pore of the cells for 48 h and further incubated for 4 h at 37°C. The absorbance optical density (OD) value at 450 nm was detected using a plate reader (Thermo Labsystems, Rochester, NY). The experiment was performed in triplicate.

### Western blot analysis

Total proteins of cells were extracted and protein concentration was measured by a BCA kit (Beyotime, China). The proteins were equally separated by 10% sodium dodecyl sulfate/polyacrylamide gel electrophoresis (SDS/PAGE) and transferred to polyvinylidene difluoride membranes (Millipore, Bedford, U.S.A.), which were then blocked by 5% skimmed milk for 1 h. Subsequently, the membranes were further incubated with the following primary antibodies (Abcam, U.S.A.): FAT4 antibody (ab130076; 1:1000; 48 kDa), VEGF antibody (ab1316; 1:1000; 21 kDa), E-Cadherin antibody (ab40772; 1:10000; 97 kDa), N-Cadherin antibody (ab18203; 1:1000; 130 kDa). Vimentin antibody (ab92547; 1:1000; 54 kDa). GAPDH antibody (ab8245; 1:1000; 36 kDa) was an internal reference. Then horseradish peroxidase-conjugated secondary antibodies (Beyotime, China) was used to incubate the membranes at 37°C for 1 h. The Image-Pro Plus 6.0 software (Media Cybernetics, Inc., Rockville, MD, U.S.A.) was used to detect the values of the protein bands.

### Scratch test

After transfection for 24 h, the cells (1 × 10^6^ cells/well) were seeded into six-well plates. Wounds were created using a 200-μl pipette on the bottom of the plate. When the cells reached 80% confluence, PBS was used to remove cell debris, and RPMI 1640 medium containing 10% FBS was replaced by fresh serum-free medium. Subsequently, the width of scratch was calculated, and scratch closure was photographed and measured under a microscope at 0 and 48 h. For statistical analysis, the data of the width of the scratches were shown as relative migration rate.

### Transwell assay

The transfected cells were suspended in the serum-free RPMI 1640 medium, and the density was adjusted to 1 × 10^5^/well. Then the cells were added to the upper chamber pre-coated with Matrigel (BD Bioscience, Woburn, MA, U.S.A.), while the lower chamber contained FBS (10%, 500 μl). After incubation for 48 h, the non-invading cells were removed from the upper chamber membrane, and the invading cells were fixed by methanol. Next, the cells were stained by 0.1% Crystal Violet. Images were taken and invasion cells were counted under a light microscope.

### Dual-luciferase reporter assay

TargetScan database (http://www.targetscan.org/vert_71/) predicted that miR-106b-5p and miR-103a-3p bind to the 3′-untranslated region (3′-UTR) site of FAT4. The wildtype (wt) FAT4-3′-UTR was amplified by PCR and cloned into the pMIR-report plasmid (Ambion, Austin, TX, U.S.A.). The mutant (mut) LASP-1 3′-UTR containing substitution nucleotides in the miR-106b-5p or miR-103a-3p target sequence was generated using the QuikChange Site-Directed Mutagenesis Kit (Stratagene, La Jolla, CA, U.S.A.). The cells were co-transfected with FAT4-3′-UTR or FAT4-3′-UTR mut and miR-106b-5p mimic or miR-103a-3p mimic or blank vectors. The transfection was performed according to instructions of Lipofectamine 2000 (Invitrogen, Carlsbad, CA). Luciferase activity was measured 48 h after transfection using dual-luciferase reporter assay system (Promega, Madison, U.S.A.).

### Statistical analysis

The data analysis was performed using SPSS 20.0 software (SPSS Inc., Chicago, IL). The data were shown as mean ± standard deviation. Student’s *t* test was used for analyzing the differences between two groups, while for differences among three or more groups, one-way ANOVA was performed. All the statistics were subjected to ANOVA followed by Bonferroni test. *P*<0.05 was considered to be a statistically significant difference.

## Results

### Expression of FAT4 in CRC tissues and CRC cell lines

**Figure 1:**
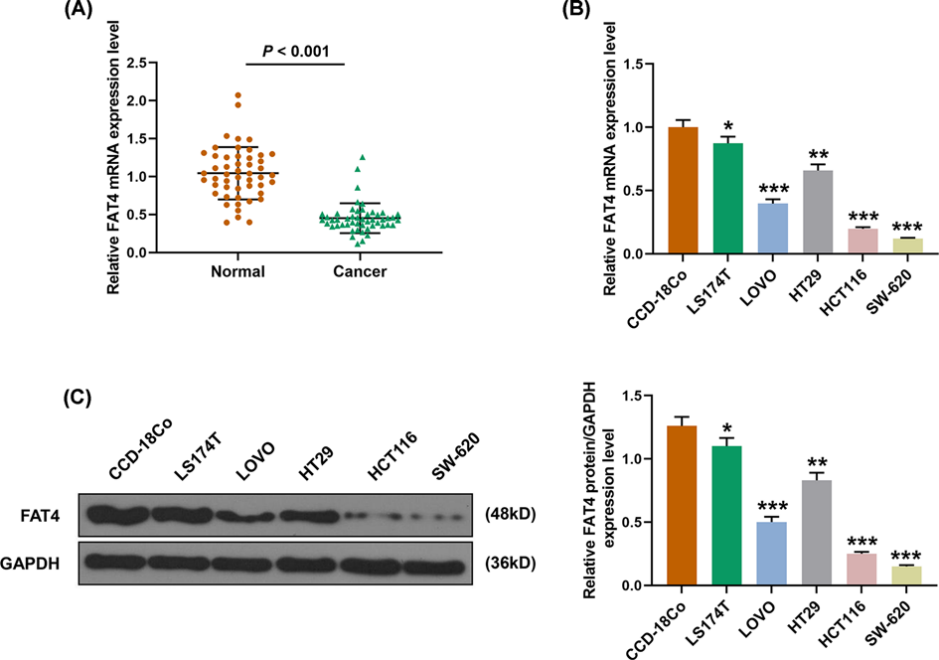
The expression of FAT4 in CRC tissues and cells (**A**) The expression of FAT4 in the cancer tissues was lower than that in normal tissues by qRT-PCR. *P*<0.001. (**B**) FAT4 mRNA was low-expressed in CRC lines than that in human normal CCD-18Co cells. (**C**) The FAT4 protein expression was lower in CRC lines than that in human normal colon cell line CCD-18Co by WB. *P<0.0.5, **P<0.01, ***P<0.001, vs. CCD-18Co.

Quantitative real-time PCR (qRT-PCR) showed that the mRNA expression level of FAT4 was down-regulated in CRC tissues compared with normal adjacent tissues (Figure 1A). qRT-PCR and Western blot (WB) assays revealed that compared with human normal colon cell line CCD-18Co, mRNA and protein expressions of FAT4 were decreased in CRC cell lines LS174T, LOVO, HT29, HCT116 and SW-620. The expression of FAT4 was the highest in LS174T, and the effect of FAT4 silencing on the cells was also observed, moreover, the lowest expression was found in SW-620 cell line, and the effect of overexpression of FAT4 on the cells was also observed (Figure 1B,C).

### The function of FAT4 silencing in the biological behaviors of LS174T

**Figure 2:**
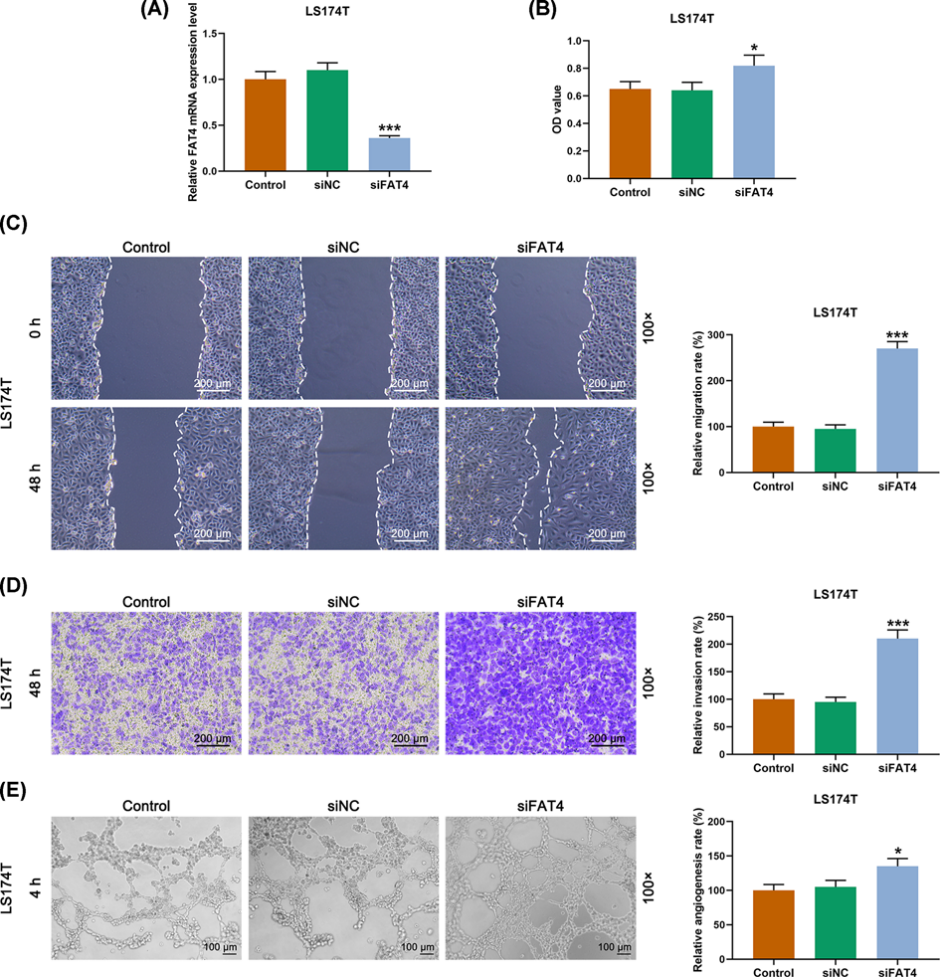
The effects of FAT4 siRNA on LS174T cell migration, invasion and angiogenesis (**A**) Successful transfection detected by qRT-PCR. (**B**) The results of CCK-8 revealed that the LS174T cell viability was increased transfected with FAT4 siRNA compared with the cells transfected with siNC. (**C**) After 48 h of cell scratch test, the scratch width in siFAT4 group was narrower than that in siNC group. (**D**) The results of transwell assay revealed that the LS174T cells transfected with FAT4 siRNA had a higher invasion rate compared with cells transfected with siNC. (**E**) The results of angiogenesis experiment showed that LS174T cells transfected with FAT4 siRNA had higher angiogenesis rate compared with that in siNC group. *P<0.5, ***P<0.001, vs. siNC.

CCK-8 showed that after the successful transfection with FAT4 siRNA (Figure 2A), the cell viability was increased by down-regulation of FAT4 in LS174T cell as compared with that in siNC group (Figure 2B). Moreover, the scratch test showed that migration of LS174T cell transfected with FAT4 siRNA was increased than that in siNC group (Figure 2C). Similar to the tendency of cell viability and migration, cell invasion and angiogenesis of LS174T cell transfected with FAT4 siRNA were higher as compared with those in siNC group (Figure 2D,E).

### The function of FAT4 silencing in biological behaviors of SW-620 cells

**Figure 3:**
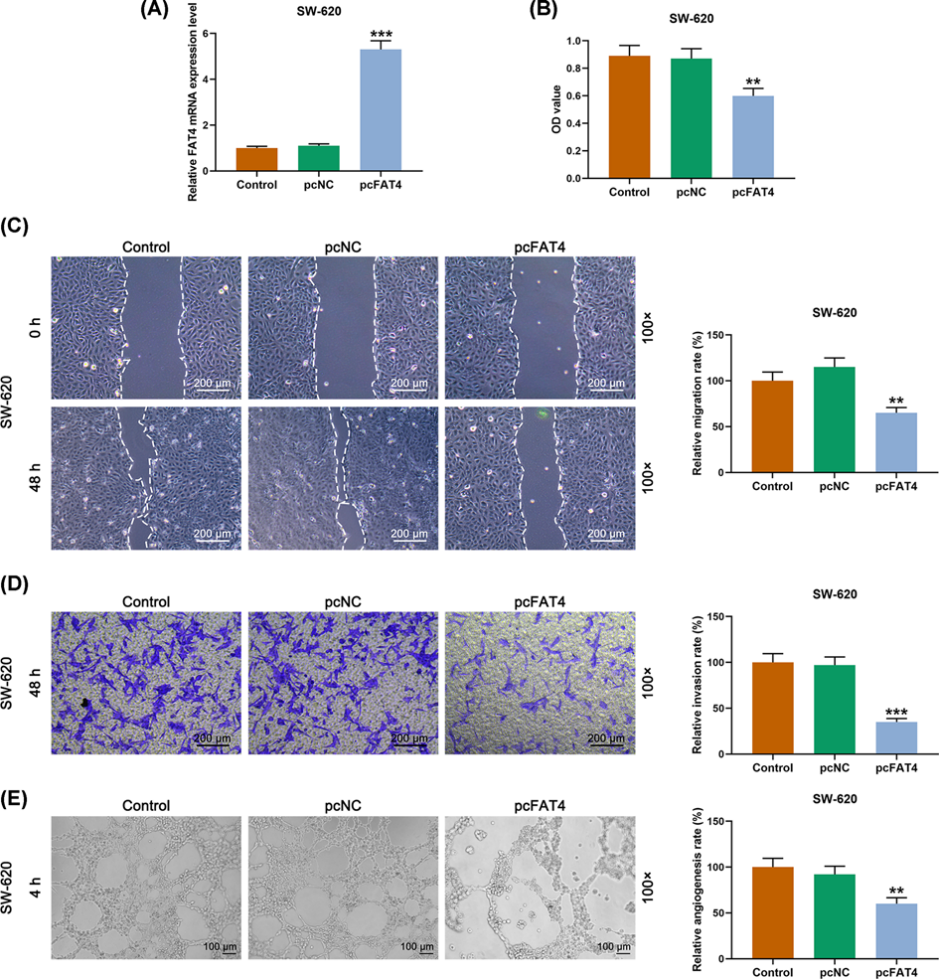
The effect of FAT4 siRNA on SW-620 cell migration, invasion and angiogenesis (**A**) Successful transfection detected by qRT-PCR. (**B**) The SW-620 cells in pcFAT4 group had lower viability compared with cells transfected with pcNC by CCK-8. (**C**) The results of cell scratch test revealed that the scratch width in pcFAT4 group was wider than that in pcNC group. (**D**) The results of transwell assay revealed that the SW-620 cell in pcFAT4 group had lower invasion rate compared with pcNC group. (**E**) SW-620 cell in pcFAT4 group had lower angiogenesis rate compared with that in pcNC group by angiogenesis experiment. **P<0.01, ***P<0.001, vs. pcNC.

CCK-8 showed that after the successful transfection of pc-DNA3.1-FAT4 plasmid into SW-620 cells (Figure 3A), the cell viability was decreased by up-regulation of FAT4 in SW-620 cells compared with that in pcNC group (Figure 3B). The scratch test revealed that the scratch width in the pcFAT4 group was wider than that in pcNC group (Figure 3C). Moreover, similar to the results of cell viability, SW-620 cell transfected with pc-DNA3.1-FAT4 plasmid showed lower cell invasion and angiogenesis rates (Figure 3D,E).

### The relation between FAT4 and miR-106b-5p

**Figure 4:**
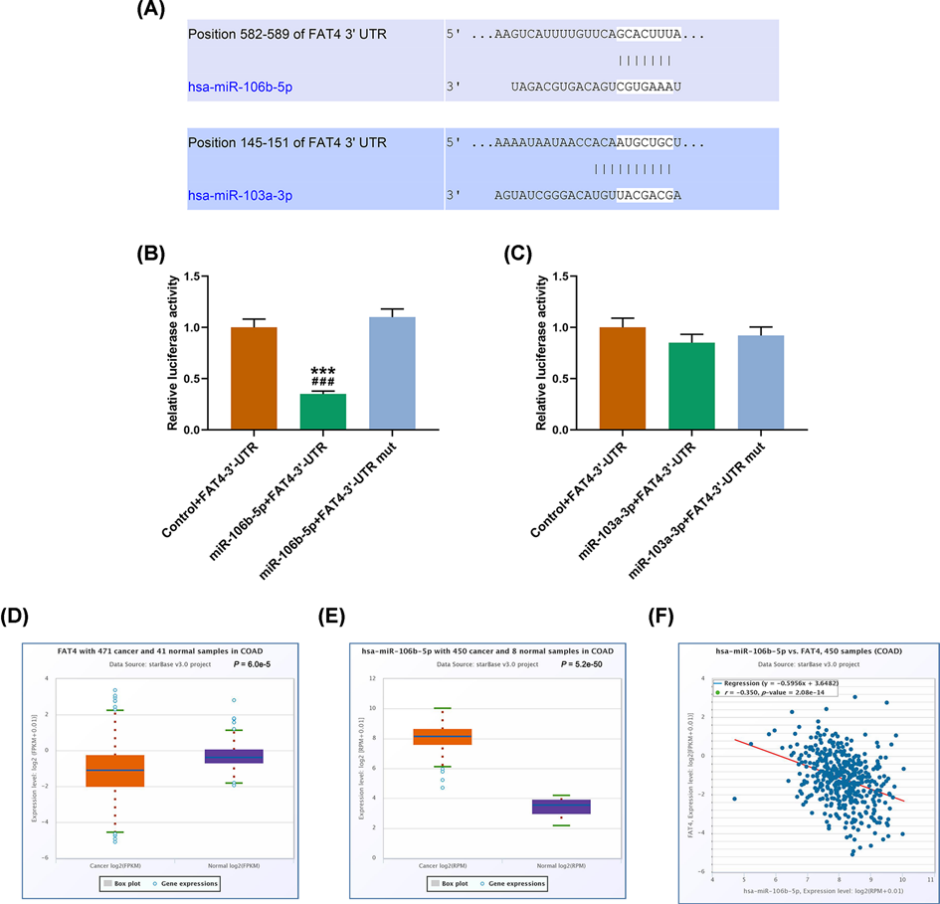
The relation between FAT4 and miR-106b-5p (**A**) Prediction of binding sites of miR-106b-5p or miR-103a-3p and FAT4 by TargetScan database (http://www.targetscan.org/vert_71/). (**B,C**) Dual-luciferase reporter assay confirmed that miR-106b-5p could inhibit the expression of FAT4, while no significant difference was found in miR-106b-5p with FAT4-mut. (**D**–**F**) TCGA analysis showed that FAT4 was low-expressed in cancer tissues and miR-106b-5p was high-expressed in colon adenocarcinoma cancer tissues, and there was a negative correlation between FAT4 and miR-106b-5p. ***P<0.001 vs. Control+FAT4-3′-UTR, ^###^*P*<0.001 vs. miR-106b-5p+FAT4-3′-UTR mut.

Prediction of binding sites of miR-106b-5p or miR-103a-3p and FAT4 were predicted by TargetScan database (http://www.targetscan.org/vert_71/) (Figure 4A). The results of dual-luciferase reporter assay (Figure 4B, C) showed that the luciferase activity of the cells co-transfected with FAT4-wt and miR-106b-5p mimic was remarkably low, while no significant difference was observed in miR-106b-5p with FAT4-mut (Figure 4B). The data from TCGA revealed that FAT4 was low-expressed and miR-106b-5p was high-expressed in CRC tissues, and StarBase (http://starbase.sysu.edu.cn) showed that FAT4 was negatively correlated with miR-106b-5p (Figure 4D–F).

### The function of miR-106b-5p in cell biological behaviors of LS174T by targeting FAT4

**Figure 5:**
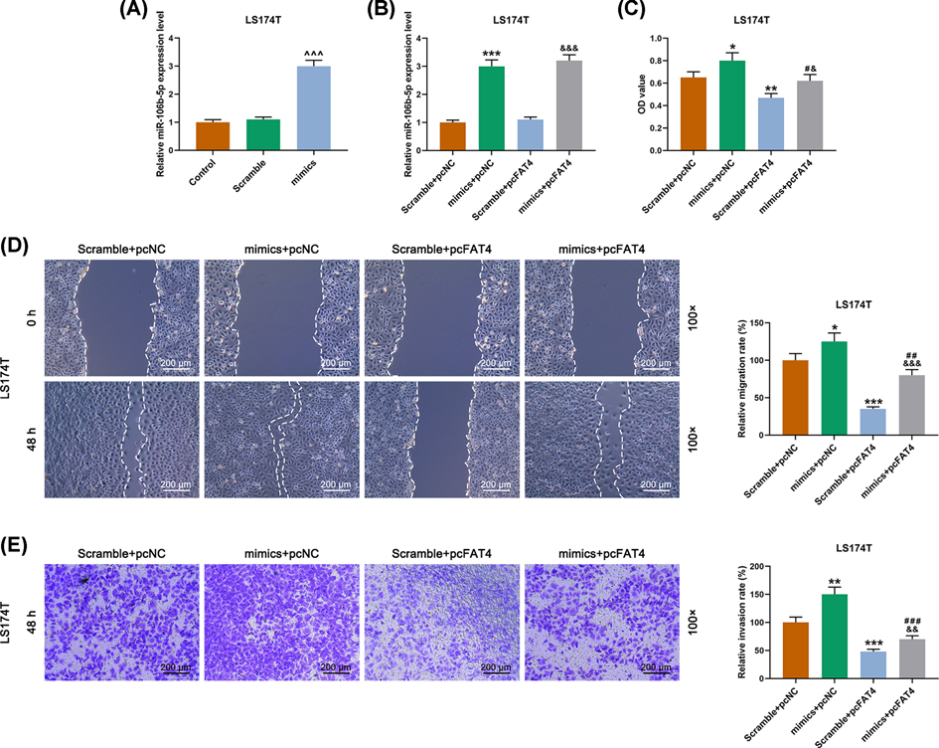
The effect of miR-106b-5p on LS174T cell proliferation, migration and invasion by targeting FAT4 (**A**) Successful transfection detected by qRT-PCR. (**B**) MiR-106b-5p mimic increased the expression of miR-106b-5p, and FAT4 overexpression had no significant effect on the expression of miR-106b-5p. (**C**) The results of CCK-8 revealed that miR-106b-5p mimic promoted cell viability, but FAT4 overexpression inhibited cell viability and significantly reversed the effect of miR-106b-5p mimics. (**D**) The results of cell scratch test revealed that miR-106b-5p mimic promoted cell migration, but FAT4 overexpression inhibited cell migration and significantly reversed the role of miR-106b-5p mimic. (**E**) The results of transwell assay revealed that miR-106b-5p mimic promoted cell invasion, FAT4 overexpression inhibited cell migration and remarkably reversed the effect of miR-106b-5p mimic. ^∧∧∧^*P*<0.001 vs. Scramble, *P<0.05, **P<0.01, ***P<0.001 vs. Scramble+pcNC, ^#^*P*<0.05, ^##^*P*<0.01, ^###^*P*<0.001 vs. mimics+pcNC, ^&^*P*<0.05, ^&&^*P*<0.01, ^&&&^*P*<0.001 vs. Scramble+pcFAT4.

**Figure 6:**
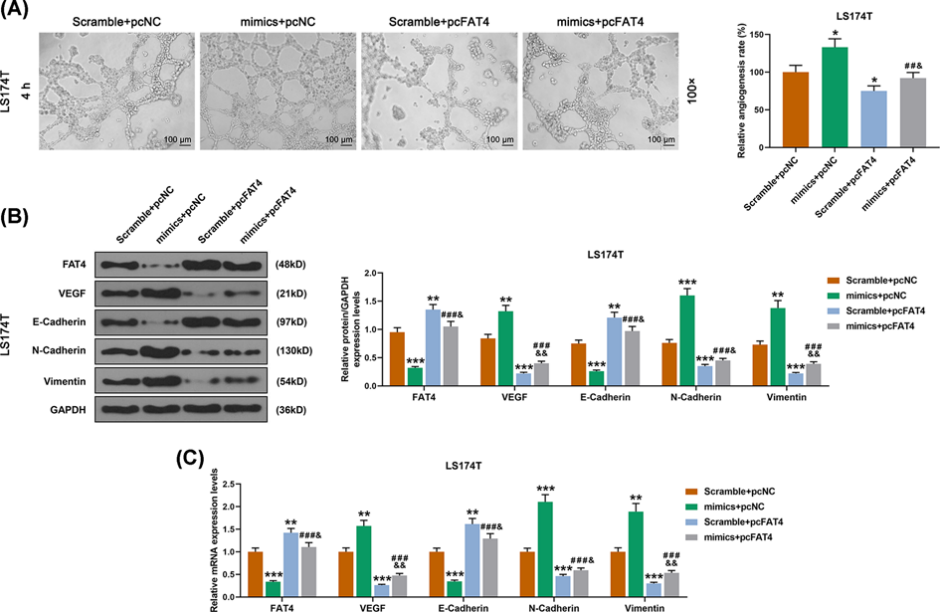
The effect of miR-106b-5p on angiogenesis of LS174T cells and relative proteins by targeting FAT4 (**A**) The results of angiogenesis experiment showed that miR-106b-5p mimic promoted angiogenesis, FAT4 overexpression inhibited cell angiogenesis and significantly reversed the effects of miR-106b-5p mimic. (**B**) WB analysis showed that miR-106b-5p mimic promoted the expressions of VEGF, N-Cadherin and Vimentin proteins; FAT4 up-regulation inhibited the expressions of those proteins, and significantly reversed the effects of miR-106b-5p mimic. (**C**) The results of qRT-PCR showed that miR-106b-5p mimic promoted the expressions of VEGF, N-Cadherin and Vimentin mRNAs, FAT4 up-regulation inhibited the expression of those mRNAs, and significantly reversed the effect of miR-106b-5p mimic. *P<0.05, **P<0.01, ***P<0.001 vs. Scramble+pcNC, ^##^*P*<0.01, ^###^*P*<0.001 vs. mimics+pcNC, ^&^*P*<0.05, ^&&^*P*<0.01 vs. Scramble+pcFAT4.

qRT-PCR showed that LS174 cells were successfully transfected (Figure 5A). The expression of miR-106-5p was increased by cell transfection with miR-106b-5p mimic, but no significant change was observed in overexpression of FAT4 (Figure 5B). According to the detection of CCK-8 assay, we found that up-regulation of miR-106b-5p promoted cell viability, whereas the opposite effects were observed in up-regulation of FAT4 (Figure 5C). In addition, the scratch test revealed that 48 h after the scratching, the scratch width was narrower in the group in which the cells transfected with miR-106b-5p mimic than those transfected with the scramble. Moreover, the up-regulation of FAT4 partially reversed the effect of miR-106b-5p overexpression (Figure 5D). The transwell assay showed that miR-106b-5p overexpression in LS174 cell had increased invasion, whereas the up-regulation of FAT4 showed opposite effects (Figure 5E). Similarly, in the angiogenesis experiment, we found that miR-106b-5p overexpression in LS174 cell increased its angiogenesis, whereas the up-regulation of FAT4 had the opposite effect (Figure 6A). The results of WB assay revealed that miR-106b-5p overexpression in LS174 cell increased the expressions of VEGF, N-cadherin, vimentin proteins and decreased the proteins expressions of FAT4 and E-cadherin in LS174 cell, whereas up-regulation of FAT4 showed the opposite effects (Figure 6B,C). Moreover, qRT-PCR revealed that up-regulation of miR-106b-5p increased the expressions of VEGF, N-cadherin, vimentin and decreased the mRNAs expressions of FAT4 and E-cadherin mRNAs in LS174 cell, whereas up-regulation of FAT4 partially reversed the effects of miR-106b-5p overexpression (Figure 6B,C).

### The function of miR-106b-5p in the biological behaviors of SW-620 cell by targeting FAT4

**Figure 7:**
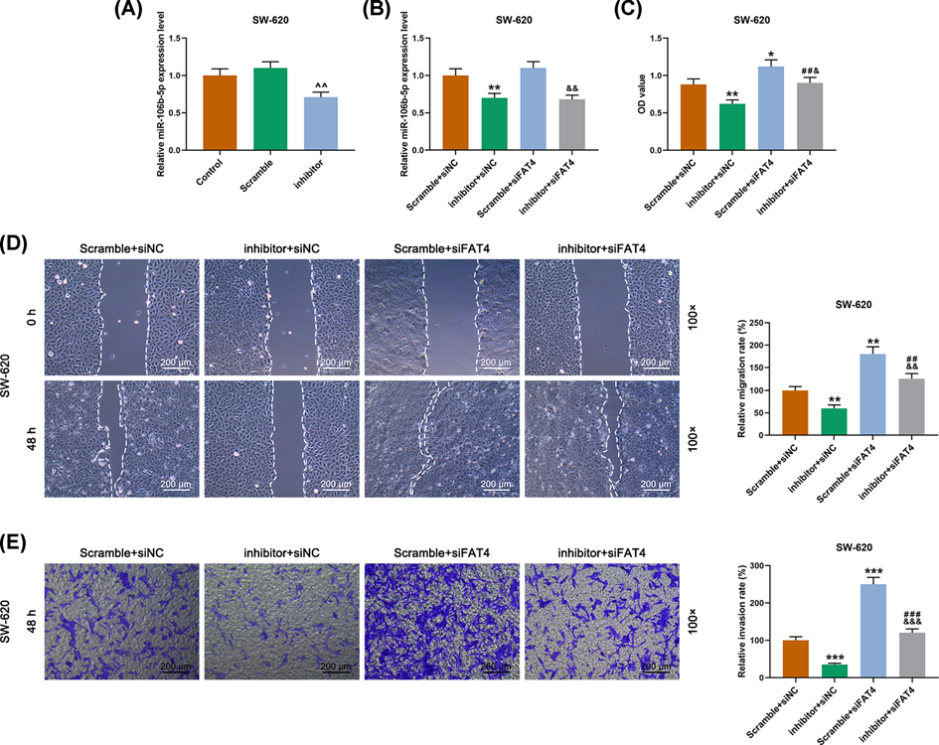
The effect of miR-106b-5p on SW-620 cell proliferation, migration and invasion by targeting FAT4 (**A**) Successful transfection detected by qRT-PCR. (**B**) MiR-106b-5p inhibitor inhibited the expression of miR-106b-5p, and FAT4 siRNA had no significant effect on its expression. (**C**) The results of CCK-8 revealed that miR-106b-5p inhibitor inhibited cell viability, siFAT4 remarkably reversed the effect of miR-106b-5p inhibitor. (**D**) The results of cell scratch test revealed that miR-106b-5p inhibitor inhibited cell migration, siFAT4 significantly reversed the role of miR-106b-5p inhibitor. (**E**) The results of transwell assay revealed that miR-106b-5p inhibitor inhibited cell invasion, siFAT4 remarkably reversed the role of miR-106b-5p inhibitor. ^∧∧^*P*<0.01 vs. Scramble, *P<0.05, **P<0.01, ***P<0.001 vs. Scramble+siNC, ^##^*P*<0.01, ^###^*P*<0.001 vs. inhibitor+siNC, ^&^*P*<0.05, ^&&^*P*<0.01, ^&&&^*P*<0.001 vs. Scramble+siFAT4.

**Figure 8:**
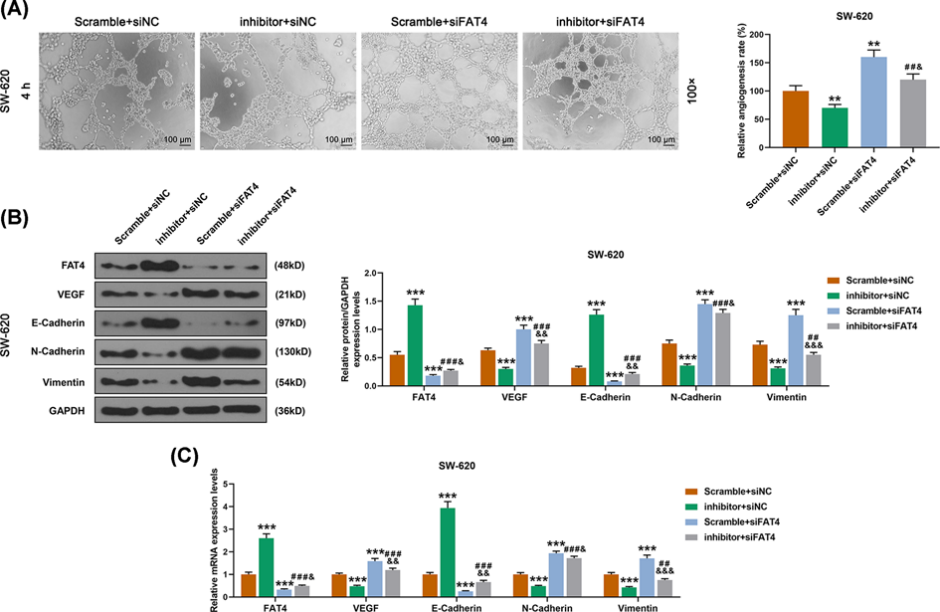
The effect of miR-106b-5p on angiogenesis of SW-620 cells and relative proteins by targeting FAT4 (**A**) The results of angiogenesis experiment showed that miR-106b-5p inhibitor inhibited angiogenesis, siFAT4 promoted cell angiogenesis and remarkably reversed the role of miR-106b-5p mimic. (**B**) The results of WB analyze showed that miR-106b-5p inhibitor inhibited the expressions of VEGF, N-Cadherin and Vimentin proteins, siFAT4 remarkably reversed the effect of miR-106b-5p inhibitor. (**C**) The results of qRT-PCR showed that miR-106b-5p inhibitor inhibited the expressions of VEGF, N-Cadherin and Vimentin mRNAs, siFAT4 significantly reversed the effect of miR-106b-5p inhibitor. **P<0.01, ***P<0.001 vs. Scramble+siNC, ^##^*P*<0.01, ^###^*P*<0.001 vs. inhibitor+siNC, ^&^*P*<0.05, ^&&^*P*<0.01, ^&&&^*P*<0.001 vs. Scramble+siFAT4.

**Figure 9:**
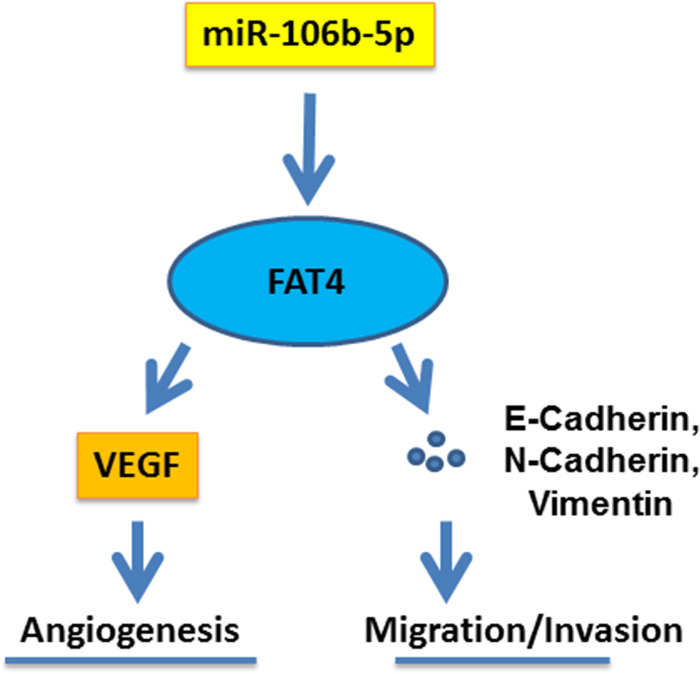
The mechanism schematic figure

The successful transfection in SW-620 cells was detected by qRT-PCR (Figure 7A). MiR-106b-5p inhibitor inhibited miR-106b-5p expression, while FAT4 silencing did not affect the expression of miR-106b-5p (Figure 7B). The results of CCK-8 assay demonstrated that down-regulation of miR-106b-5p inhibited cell viability, whereas the FAT4 silencing partially reversed the effect of down-regulation of miR-106b-5p (Figure 7C). Moreover, the scratch test and the transwell assay showed that down-regulation of miR-106b-5p inhibited cell migration and invasion, whereas the FAT4 silencing showed the opposite effects (Figure 7D,E). In addition, the result of angiogenesis experiment revealed that down-regulation of miR-106b-5p inhibited the angiogenesis of SW-620 cells, whereas FAT4 silencing partially reversed the effect of down-regulation of miR-106b-5p (Figure 8A). Furthermore, the results of WB and qRT-PCR analysis revealed that the proteins and mRNAs expressions of VEGF, N-cadherin, vimentin were decreased and those of FAT4 and E-cadherin proteins and were increased in SW-620 cells transfected with miR-106b-5p inhibitor, while the FAT4 silencing partially reversed the effect of down-regulation of miR-106b-5p (Figure 8B,C). The mechanism schematic figure of miR-106b-5p targeting on FAT4 in CRC was shown in Figure 9.

## Discussion

CRC is a frequent cancer and the fourth leading cause of cancer-related mortality, and its incidence is increasing annually [18]. Our study revealed that miR-106b-5p targeting FAT4 had an anti-cancerous effect on CRC by regulating the epithelial–mesenchymal transition (EMT) process, which may provide a theoretical reference for the precise treatment of CRC.

Our results indicated that the FAT4 was low-expressed in CRC tissues than the adjacent tissues. A similar result was found in the cell experiment in which FAT4 was low-expressed in CRC cell lines than the normal colon cell. A study indicated that FAT4 has a low expression in gastric cancer and is targeted by miR-107, which results in activation of PI3K/AKT signaling and causes the promotion of gastric cancer growth and metastasis [19]. Moreover, down-regulation of FAT4 in CRC cells inhibited the cell viability, migration, invasion and angiogenesis rates, however, overexpression of FAT4 promoted CRC viability, and increased cell migration, invasion, and angiogenesis. A previous study [20] reported that FAT4 expression is remarkably decreased in gastric cancer tissues and is lower than that in non-cancerous adjacent tissues, which causes Yap nuclear translocation, noticeably, such a mechanism of FAT4 is correlated with poor prognosis, that is to say, tumor infiltration, lymph node metastasis and lower cumulative survival. Therefore, we speculated that FAT4 may has an anti-cancerous function in the proliferation and metastasis of some cancers, and regulating FAT4 expression may become one of the new directions of cancer treatment.

MiRNAs are endogenous non-coding RNAs composed of 19–24 nucleotides, and bind to the 3′-UTR complementary site of target mRNAs to regulate the expressions of target genes and further inhibit translation and degrade target genes, thereby regulating cell differentiation, proliferation, apoptosis and DNA repair [21–23]. In this research, we found that in the CRC tissues the expression of miR-106b-5p was increased and was higher than that in normal tissues. The dual-luciferase reporter assay verified the target relation between FAT4 and miR-106b-5p, and rescue experiment revealed that up-regulation of miR-106b-5p expression promoted the viability and increased migration, invasion and angiogenesis of the cells, and overexpression of FAT4 reversed the function of up-regulation of miR-106b-5p in CRC cells, however, low-expressed miR-106b-5p showed the opposite effects on CRC cells via targeting FAT4. According to a previous study [24], miR-106b-5p promotes malignant melanoma progression possibly through targeting PTEN to regulate cell cycle-related proteins and Akt/ERK signaling pathway. MiR-106b-5p plays a carcinogenic role in hepatocellular carcinoma through regulating the target gene *RUNX3* [25]. In addition, a recent clinical study incorporating 284 renal carcinoma patients showed that the expression of miR-106b-5p is negatively correlated with the overall survival of the patients, moreover, the study also indicated that miR-106b-5p expression was an independent predictor of poor prognosis in renal carcinoma patients [26]. Thus, miR-106b-5p plays a carcinogenic role in a variety of cancers through different regulatory mechanisms.

The current findings showed that FAT4 was negatively correlated with miR-106b-5p by using starBase website. The CRC cells were transfected with FAT4 overexpression or silencing in combination with miR-106b-5p mimics or inhibitor to study the relationship of miR-106b-5p and FAT4 on the migration and invasion of CRC cells.

In addition, the results of WB analysis showed the regulatory effects of miR-106b-5p on viability, migration, invasion and angiogenesis of CRC cells via targeting FAT4 was realized through regulating the EMT progress. EMT is considered as a major driver of tumor metastasis, and is closely associated with a variety of metastasis and invasion of cancer [27–29]. Previous reports showed that the low expression of dual oxidase 1 may be an indicator of poor prognosis of lung cancer, and its high expression in lung cancer cells can reverse EMT and enhance the characteristics of epithelial cells [30]. Study [31] also found that tissue inhibitors of metalloproteinase 2, a multifunctional protein, inhibits tumor growth, invasion and metastasis through EMT. In our research, we found that in CRC cells the decreased expression of miR-106b-5p, the inhibition of the expression of angiogenesis factor VEGF, reversed EMT process through increasing the expressions of E-cadherin and decreasing N-cadherin and Vimentin via targeting FAT4. Collectively, our study revealed that miR-106b-5p regulated the biological behaviors of CRC cells by targeting FAT4, which may provide new understanding for the treatment of CRC. However, the present study still has some limitations, as the role of miR-106b-5p targeting FAT4 in CRC was not further validated in *in vivo* animal model and other possible mechanisms involved were not investigated. Moreover, it is also another limitation not studying miR-106-5p-dependent modulation of FAT4 in CRC under endogenous conditions.

In conclusion, the present findings demonstrated the differences in miR-106b-5p and FAT4 expressions in CRC tissues, and revealed that miR-106b-5p played a carcinogenic role in the CRC cells by targeting FAT4. The mechanism may be related to the regulation of angiogenesis factors and EMT process.

## Abbreviations

CCK-8: cell counting kit-8
CRC: colorectal cancer
EMT: epithelial–mesenchymal transition
FAT4: FAT atypical cadherin 4
FBS: fetal bovine serum
miRNA: microRNA
GAPDH: glyceraldehyde-3-phosphate dehydrogenase
mRNA: messenger RNA
pcNC: pc DNA3.1-NC
qRT-PCR: quantitative real-time PCR
TCGA: The Cancer Genome Atlas
VEGF: vascular endothelial growth factor
WB: Western blot
