# Expression of Concern: MiR-106b-5p Regulates the Migration and Invasion of Colorectal Cancer Cells by Targeting FAT4

**DOI:** 10.1042/BSR-20200098_EOC

**Published:** 2021-04-07

**Keywords:** Colorectal cancer, FAT4, MicroRNA-106b-5p

The Editorial Office has been made aware of potential issues surrounding the scientific validity of this paper, hence has issued an expression of concern to notify readers whilst the Editorial Office investigates.

